# Prebiotic Systems Containing Anthocyanin-Rich Pomegranate Flower Extracts with Antioxidant and Antidiabetic Effects

**DOI:** 10.3390/pharmaceutics16040526

**Published:** 2024-04-10

**Authors:** Anna Gościniak, Natalia Rosiak, Daria Szymanowska, Andrzej Miklaszewski, Judyta Cielecka-Piontek

**Affiliations:** 1 Department of Pharmacognosy and Biomaterials, Poznan University of Medical Sciences, Rokietnicka 3, 60-806 Poznan, Poland; anna.gosciniak@student.ump.edu.pl (A.G.); nrosiak@ump.edu.pl (N.R.); daria.szymanowska@up.poznan.pl (D.S.); 2 Department of Biotechnology and Food Microbiology, Poznan University of Life Sciences, Wojska Polskiego 48, 60-627 Poznan, Poland; 3 Institute of Materials Science and Engineering, Poznan University of Technology, Jana Pawła II 24, 61-138 Poznan, Poland; andrzej.miklaszewski@put.poznan.pl

**Keywords:** pomegranate flower extract, anthocyanins, cyclodextrins, anti-diabetic activity, prebiotic potential

## Abstract

Pomegranate flower extract, rich in anthocyanins, demonstrates beneficial health-promoting properties such as an anti-diabetic and antioxidant effect, among others. However, the potential health-promoting properties may be hindered by the low stability of anthocyanins. Therefore, the aim of our study was to assess whether stabilizing carriers, namely HP-γ-cyclodextrin (HP-γ-CD), α-cyclodextrin (α-CD), Methyl-β-cyclodextrin (Me-β-CD), Inulin (Inu) and Arabic gum (AGu) affect the antioxidant and antidiabetic activity of lyophilized pomegranate flower extract, how they influence stability, release profile, and whether the systems exhibit prebiotic activity. Interactions between pomegranate flower extract and these factors were analyzed using FT-IR. The structures were examined through microscopic imaging while for the prepared prebiotic systems, antidiabetic activity was determined and confirmed by the inhibition of α-amylase and α-glucosidase; antioxidant activity was expressed by DPPH and CUPRAC assays. The content of pelargonidin-3,5-glucoside in these systems was assessed using the HPLC method. The release profiles of pelargonidin-3,5-glucoside were examined in a medium at pH = 6.8 and pH = 1.2, and the stability was assessed after subjecting the systems to high temperatures (T = 90 °C). The prebiotic potential was evaluated for 10 prebiotic bacterial strains (*Lactobacillus acidophilus*, *Lactobacillus casei*, *Lactobacillus plantarum*, *Lactobacillus brevis Lactobacillus rhamnosus gg*, *Lactobacillus reuteri*, *Pediococcus pentosaceus*, *Lactococcus lactis*, *Lactobacillus fermentum lf*, *Streptococcus thermophilus*). As a result of the conducted research, better functionalities of the obtained systems containing Pomegranate flower extract were proven in terms of prebiotic and antidiabetic effects. The obtained delivery systems for pelargonidin-3,5-glucoside allow for better use of its health-promoting effects.

## 1. Introduction

Anthocyanins are a class of natural compounds found in various fruits, vegetables, and plants, known for their potential health benefits, including their possible role in managing diabetes [1]. These compounds have been of particular interest due to their antioxidant and anti-inflammatory properties, which could contribute to improved glucose regulation and insulin sensitivity [2,3]. In vitro studies have shown that anthocyanins can reduce the activity of α-glucosidase and α-amylase enzymes by directly interacting with them. The mechanism involves the formation of complexes with the enzymes, leading to a reduction in their catalytic activity. These findings suggest that anthocyanins may play a role in controlling blood glucose levels and managing the risk of diabetes and metabolic diseases [4,5]. However, despite their potential advantages, anthocyanins come with a notable limitation: their low stability [6]. Stability refers to the ability of a compound to maintain its chemical structure and properties over time, especially when exposed to various environmental conditions. In the case of anthocyanins, their molecular structure makes them susceptible to degradation, leading to a decrease in their potency and effectiveness [7,8,9].

Pelargonidin-3,5-O-glucoside is an anthocyanin found in a variety of natural sources, one of which is the pomegranate flowers. Pelargonidin-3,5-O-glucoside, like other anthocyanins, influences the coloration of flowers and fruits, but it also possesses health-promoting activity by exhibiting antioxidant and anti-inflammatory activity [10,11]. Studies have explored the presence of pelargonidin-3,5-O-glucoside in different plant species, such as the family of Vitaceae [12]. Analytical techniques, like HPLC and mass spectrometry, have facilitated the identification and quantification of this anthocyanin in various plant sources [13].

The prebiotic effect in diabetes is also vital for maintaining gut health and potentially influencing blood sugar control. Prebiotics, which serve as nourishment for beneficial gut bacteria, have been shown to impact glucose metabolism and regulation. Dysbiosis, marked by alterations in microbiota diversity and composition, has been linked to the disruption of metabolic activities, leading to the onset of obesity and diabetes. Research suggests that prebiotics may enhance insulin sensitivity and reduce inflammation, both important factors in diabetes management. Additionally, prebiotics could play a role in weight management and overall gut health, benefiting individuals with diabetes [14,15]. Studies suggest that prebiotics have the ability to promote the growth of beneficial gut microbes, which can influence metabolic health and balance the gut microbiota in people with diabetes. This prebiotic activity may help improve glycemic control in diabetic patients by affecting glucose metabolism and insulin resistance. In addition, it is possible that these prebiotic substances may affect the inflammatory and oxidative processes that are associated with diabetes by affecting the gut microbiota and the products of bacterial metabolism [16]. However, the addition of a properly selected excipient can create a system that will favorably influence the microbiome by showing prebiotic effects [17,18].

Oligosaccharides are commonly used as prebiotics as they promote the growth of beneficial bacteria in the gastrointestinal tract. Cyclodextrins, cyclic oligosaccharides linked by α-1,4-glycosidic bonds, although exhibiting similar beneficial properties are less frequently employed as prebiotics. These systems exhibit various advantages, including, notably, enhanced solubility. By encapsulating bioactive compounds within cyclodextrin molecules, the resulting complex exhibits improved dispersibility in aqueous solutions [19].

Cyclodextrins can be used as a prebiotic because of their properties, such as being tasteless, odorless, indigestible, calorie-free and having a low glycemic index. Cyclodextrins undergo limited enzymatic digestion within the human digestive tract, instead undergoing fermentation in the intestines, thereby promoting improvements in the intestinal microflora. These compounds demonstrate anti-obesity and anti-diabetic effects, imparting health benefits. Cyclodextrins can be applied as dietary supplements and nutraceuticals for the modulation of body weight, lipid profile, and blood sugar levels [20].

Inulin is a fructan-type polysaccharide with a unique chemical structure that plays a significant role in its properties and applications. The structure of inulin consists of (2→1) linked β-D-fructosyl residues, typically with an α-D-glucose end group, with a varying degree of polymerization ranging from 2 to 60. Inulin is a well-known prebiotic, as it acts as food for beneficial intestinal bacteria, supporting a healthy intestinal microflora [21].

**Figure 1:**
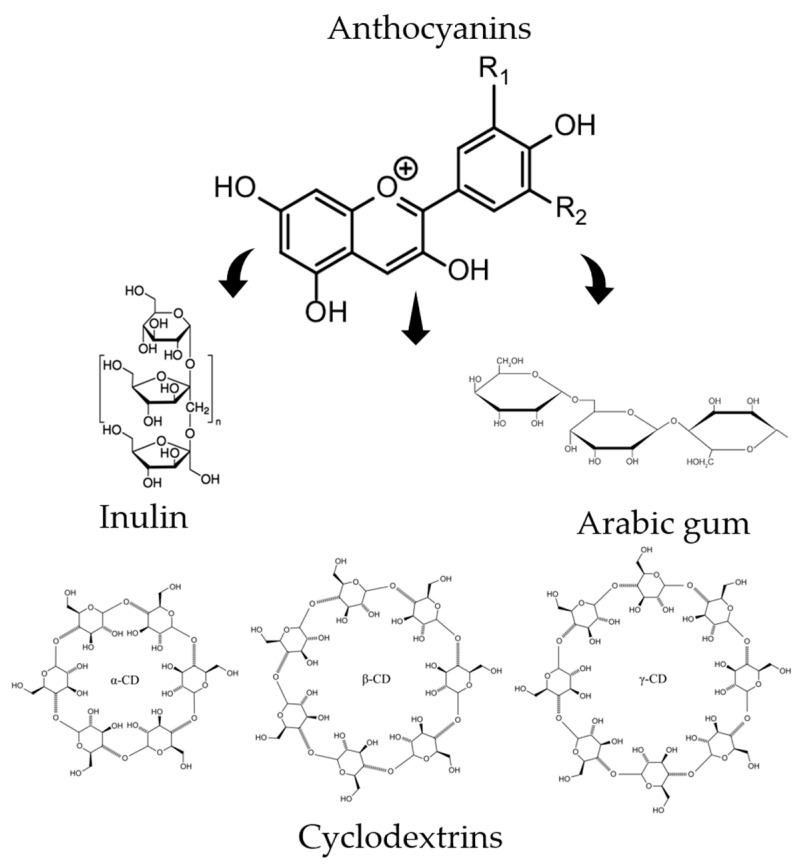
General structure of the anthocyanins and the main structures of the substances used in the study to obtain the systems.

The structure of gum Arabic consists of a heterogeneous mixture comprising highly branched polysaccharides, arabinogalactan-protein, and a glycoprotein fraction, with the polysaccharide fraction accounting for the majority of its weight [22]. Gum Arabic has also been frequently studied recently for its prebiotic properties [23]. The study suggests that gum Arabic can be classified as a prebiotic fiber, performing as effectively as inulin by promoting the growth of beneficial bacteria in larger quantities without stimulating undesirable bacterial populations [24,25]. The structure of the compounds used in the study is shown in Figure 1.

The aim of the study was to characterize systems containing pomegranate flower extract with substances of potential prebiotic character. The effect of carriers on the properties of the extract was evaluated in the context of antioxidant, anti-diabetic, and microbial activity, as well as what effect the combination has on the stability and release profile of the active compound. The model carriers chosen were HP-γ-cyclodextrin, α-cyclodextrin, methyl-β-cyclodextrin, inulin and gum Arabic.

## 2. Materials and Methods

### 2.1. System Preparation

Five grams of pomegranate flowers were extracted from an ultrasonic bath under optimal conditions in order to obtain the highest possible anti-diabetic activity based on the publication by Gosciniak et al. [26]. The extract obtained was lyophilized (LyoQuest Telstar, Barcelona, Spain) and then combined with an excipient in a 1:1 ratio, dissolved in water and lyophilized again. The systems were then unified in an agate mortar.

The following systems were obtained:

1. HP-γ-CD/PL (HP-γ-cyclodextrin/pomegranate flower lyophilized extract)
2. α-CD/PL (α-cyclodextrin/lyophilized pomegranate flower extract)
3. Me-β-CD/PL (Methyl-β-cyclodextrin/lyophilized pomegranate flower extract)
4. Inu/PL (Inulin/lyophilized pomegranate flower extract)
5. AGu/PL (Arabic gum/lyophilized pomegranate flower extract)

The microphotograph was taken by VHX 7000 Keyence microscope using digital depth composition mode at 16-bit high dynamic range.

### 2.2. HPLC Analysis

The HPLC analysis was carried out to determine pelargonidin-3,5-O-glucoside according to the methodology of Gościniak et al. [26].

### 2.3. FT-IR Analysis of Prebiotic Systems

The (ATR-FTIR) spectra were recorded on an IRTracer-100 spectrophotometer (Kyoto, Japan). The spectra were measured within the frequency range of 4000 and 400 cm^−1^ in the absorbance mode. The parameters of the apparatus were as follows: resolution, cm^−1^; number of scans, 400; apodization, Happ-Genzel. The samples were placed on the ATR crystal and pressed against it whilst the ATR-FT-IR spectrum was scanned. The spectra of RA and CDs and the physical mixtures of the systems were also analyzed. The spectra were analyzed with the use of the OriginPro 8 software (OriginLab Corporation, Northampton, MA, USA). Functional group assignment was based on the literature and is included in Table S2 (Supplementary Materials) [27,28,29,30,31,32,33,34,35,36,37,38,39,40,41,42].

### 2.4. Dissolution Study of Pelargonidin-3,5-O-glucosides

A dissolution study was performed using a paddle apparatus (Agilent Technologies, Santa Clara, CA, USA). Approximately 50 mg of PL and 100 mg of systems were accurately measured and placed in gelatin capsules. These capsules were then placed within springs to prevent them from floating. The experiment was conducted for a duration of 120 min under a pH of 1.2. The containers were filled with 500 mL of a medium containing 0.1 N HCl with a pH of 1.2. The temperature was kept constant at 37 °C ± 0.5, and the rotation speed was set to 50 rpm. At specific time intervals, 5 mL samples were withdrawn and immediately replaced with the same volume of freshly prepared medium at the desired temperature. These samples were then filtered through a 0.22 μm membrane filter and subjected to HPLC analysis. The similarity of the dissolution profiles of lyophilized extracts and systems with excipients was established using difference (*f*_1_) and similarity (*f*_2_) factors and was defined by the following equations:

$$f_{1}=\frac{\sum_{j=1}^{n}\left|R_{j}{-T}_{j}\right|}{\sum_{j=1}^{n}R_{j}}\times 100$$

$$f_{2}=50\times log\left({\left(1+\left(\frac{1}{n}\right){\sum_{j=1}^{n}|R_{j}-T_{j}|}^{2}\right)}^{-\frac{1}{2}}\times 100\right)$$

where *n* is the number of time points, *R_j_* is the percentage of released active compound from the reference sample (PL), *T_j_* is the percentage of released active compound from the tested system. Dissolution profiles are different when the *f*_1_ value is between 0 and 15 and *f*_2_ is close to 100 (between 50 and 100)

### 2.5. Kinetics of Pelargonidin-3,5-O-glucosides Degradation

In the degradation test, samples containing specific amounts of the test substance were heated in a thermostatic bath at 90 °C ± 1 °C for 240 min. During the experiment, samples were taken at intervals and cooled in ice, dissolved in water and the concentration of the test substance after dissolution was measured. The time at which the sample solution reached the set temperature could be omitted. The Pe-3,5-glu retention (%) of the different samples was compared with the initial Pe-3,5-glu content (*C*_0_) prepared for each sample. Previous studies have shown that thermal degradation of anthocyanins matched the first-order reaction kinetics. The kinetic model can be expressed by the following equations:

$$C_{t}=C_{0}e^{-kt}$$

where *C*_0_ is the initial Pe-3,5-glu contents, *C_t_* is the Pe-3,5-glu contents after a certain time (*t* min) at the given temperature, *k* is the first-order kinetic constant (min^−1^), and *t* is time (min).

To determine the half-time (*t*_1/2_), the formula was used:

$$t_{1/2}=-\ln(0.5)/k$$

where *k* is the first-order kinetic constant (min^−1^).

### 2.6. Antioxidant and Antidiabetic Activity of Lyophilizate and Systems

#### 2.6.1. DPPH Assay

The antioxidant activity test by assessing the DPPH radical scavenging capacity was carried out according to the methodology described by Studzińska-Sroka et al. [43]. The free radical scavenging capacity (%) was calculated from equilibrium:

$$DPPH\;scavenging\;activity\;(\%)=\left[{(A}_{0\;\;}-A_{1}\right)/A_{0}]\times 100\%$$

where *A*_0_ is the absorbance of the control, and *A*_1_ is the absorbance of the sample. From the results obtained, the IC_50_ value was calculated. The IC_50_ value is interpreted as a concentration that has a scavenging activity of 50%.

#### 2.6.2. CUPRAC Assay

The CUPRAC antioxidant activity test was conducted according to the methodology outlined by Studzińska-Sroka et al. [43]. The results were expressed as the IC_0.5_, which is the concentration of sample necessary to achieve an absorbance value of 0.5.

#### 2.6.3. α-Glucosidase Inhibitory Assay

Inhibition of α-glucosidase was performed according to Gościniak et al. [26]. Briefly, sample solutions, phosphate buffer (pH = 6.8), and α-glucosidase (0.5 U/mL) were mixed and preincubated at 37 °C for 15 min. Then, *p*-nitrophenyl-α-D-glucopyranoside (pNPG) was added and incubated at 37 °C for 20 min. The reaction was stopped with sodium carbonate, and the absorbance was measured at 405 nm to quantify released *p*-nitrophenol. The degree of enzyme inhibition was calculated according to the following formula:

$$Enzyme\;Inhibition\;(\%)=\left[{(A}_{0\;\;}-A_{1}\right)/A_{0}]\times 100\%$$

where *A*_0_ is the absorbance of the control reduced by the sample background (100% enzyme activity), and *A*_1_ is the absorbance of the tested sample reduced by the sample background. In order to compare the activity, the IC_50_ value was determined. The IC_50_ value is interpreted as the concentration that inhibits 50% of the enzyme activity.

#### 2.6.4. α-Amylase Inhibitory Assay

The amylase inhibition test was performed according to the methodology described by Studzińska-Sroka et al. [43] with some modifications. Enzymes and tested samples in different concentrations were pre-incubated. Then, phosphate buffer and starch solution (0.5%) were added and re-incubated. A color reagent was added, followed by incubation and cooling. Absorbance was measured at 540 nm and was expressed as a percentage using the formula:

$$Enzyme\;Inhibition\;(\%)=\left[{(A}_{0\;\;}-A_{1}\right)/A_{0}]\times 100\%$$

where *A*_0_ is the absorbance of the control reduced by the sample background (100% enzyme activity), and *A*_1_ is the absorbance of the tested sample reduced by the sample background. A blank control was a sample prepared in the absence of the starch solution. In order to compare the activity, the IC_50_ value was determined. The IC_50_ value is interpreted as the concentration that inhibits 50% of the enzyme activity.

#### 2.6.5. Molecular Docking

The X-ray crystallographic structure of α-glucosidase and α-amylase was retrieved from the Protein Data Bank (http://www.rcsb.org/pdb, accessed on 16 November 2022). The molecular structures of pelargonidin 3,5-diglucoside (Pe-3,5-glu, PubChem CID: 167642) was retrieved from PubChem (https://pubchem.ncbi.nlm.nih.gov, accessed on 16 November 2023). The geometries of Pe-3,5-glu were optimized using GaussView software (version E01, Wallingford, CT, USA) with the density functional theory (DFT) method employing Becke’s three-parameter hybrid functional (B3LYP) along with the standard 6–311G(d,p) basis set. Followed by the preparation of ligands and proteins using AutoDock Tools (ADT; Scripps Research Institute, La Jolla, San Diego, CA, USA) [44]. This preparation involved removing water and other ligands and adding polar hydrogen atoms and Kollman charges. The AutoDock Tools 4.2 (ADT; Scripps Research Institute, La Jolla, San Diego, CA, USA) [44] were used to create a grid box to incorporate the entire active site for each protein structure (Table 1) and the Lamarckian Genetic Algorithm method with 100 conformations. The grid box was centered around the active site pocket as predicted by PrankWeb (https://prankweb.cz/, accessed on 16 November 2023). Parameters used to create a grid box are included in Table 1.

Molecular docking was conducted using Autodock Vina version 1.2.0 (the Scripps Research Institute, La Jolla, San Diego, CA, USA) [45,46]. Following the docking simulations, the best scoring pose was chosen and saved in PDBQT format. This file was then converted to PDB format using the Open Babel 3.1.1 [47] and analyzed using the protein–ligand interaction profiler (PLIP server, https://plip-tool.biotec.tu-dresden.de/, accessed on 20 November 2022) [48]. The PLIP server was utilized to identify interactions between Pe-3,5-glu and the active site of the enzymes. Subsequently, a file in PSE format was obtained from the PLIP server. The docked complexes of Pe-3,5-glu/α-glucosidase and Pe-3,5-glu/α-amylase (PSE format) were visualized using the PyMOL tool 2.5.1 (DeLano Scientific LLC, Palo Alto, CA, USA) [49].

### 2.7. Microbiology Study of Prebiotic Systems

The study material consisted of strains of lactic fermentation bacteria with potential probiotic properties deposited in the microorganism collection of the Department of Food Biotechnology and Microbiology of the University of Life Sciences in Poznań. The bacteria were stored frozen at −40 °C with 20% glycerol. The bacteria used in the study were: *Lactobacillus acidophilus 4356*, *Lactobacillus casei ATCC 393*, *Lactobacillus plantarum ATCC 14917*, *Lactobacillus brevis ATCC 8287*, *Lactobacillus rhamnosus gg ATCC 53103*, *Lactobacillus reuteri ATCC 5289*, *Pediococcus pentosaceus ATCC 25745*, *Lactococcus lactis ATCC 11955*, *Lactobacillus fermentum lf 2 (lmg 27299)*, and *Streptococcus thermophilus fp 4 (dsm 18616)*.

Probiotic bacteria strains were prepared by suspending 0.1 g of lyophilized bacteria in 10 mL of MRS liquid medium and incubating at 37 °C for 18 h. After centrifugation and washing, the biomass was diluted to a concentration of 1.0 × 10^2^ cfu/mL in 0.9% NaCl. Test samples at 100 μg/mL concentration were prepared in 0.9% NaCl. These were then inoculated with the prepared bacteria suspension and incubated at 37 °C for 18 h. The number of bacteria was assessed before and after incubation.

## 3. Results and Discussion

### 3.1. Microscopic and HPLC Analysis of Obtained System

**Figure 2:**
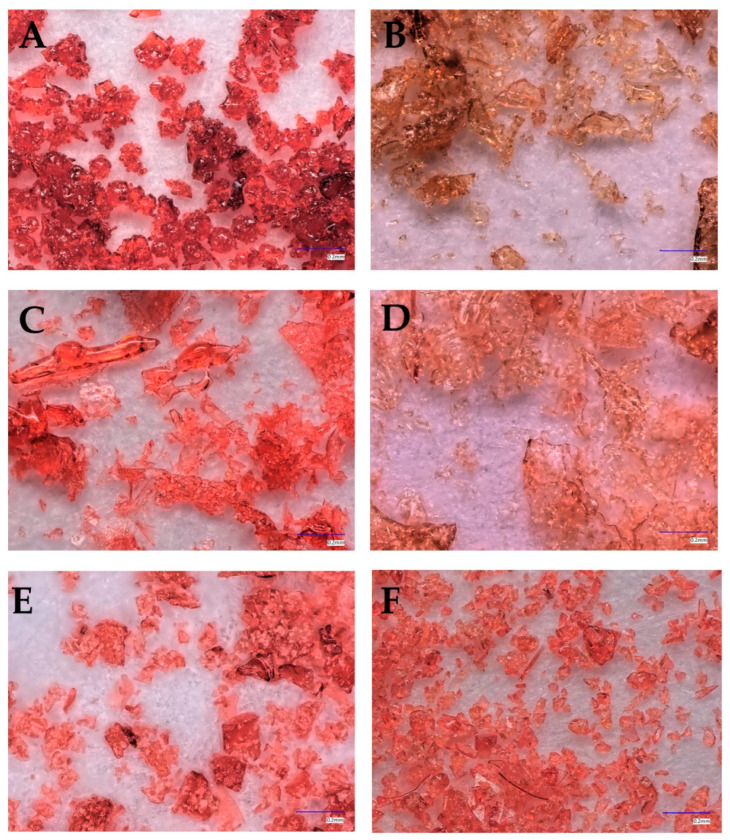
Microscopic images of PL (**A**) and the obtained systems: HP-γ-CD/PL (**B**) α-CD/PL (**C**) Me-β-CD/PL (**D**), Inu/PL (**E**), AGu/PL (**F**).

The prepared systems containing lyophilizates of extracts from pomegranate flower extract and model carriers were characterized by homogeneous extract and good flowability. The microscopic results of the systems are shown in Figure 2. Microscopic analysis of the samples revealed distinctive structural features that are key to understanding the properties and functions of the materials studied. During microscopic analysis, the studied systems were found to have a fairly homogeneous structure, indicating an even distribution of components in the material. Any inhomogeneities did not affect the homogeneity shown by HPLC.

**Figure 3:**
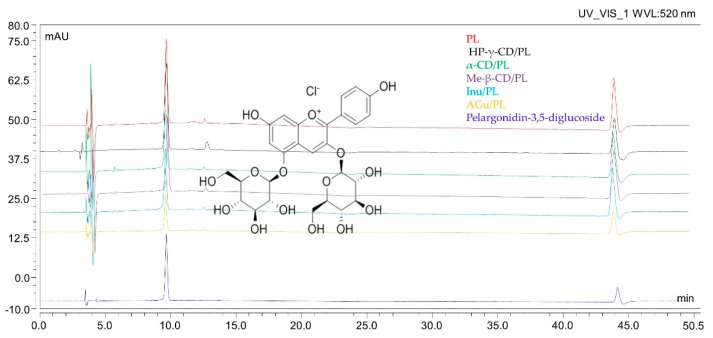
HPLC chromatograms of pomegranate flower extract and active substance standard.

Using HPLC analysis, it was determined that the main component of the anthocyanin group was pelargonidin-3,5-glycoside. While another compound belonging to the anthocyanin group, pelargonidin-3-glucoside was also present in the extract, its amount was much lower, so it was not included in the quantitative analysis. Confirmation of the pelargonidin-3,5-glycoside content was observed in the systems (Figure 3). The receipt of the systems did not result in the appearance of degradation products visible on the chromatogram. The content of the active compound per milligram of the obtained system (Table 2) is half as much as in the lyophilized extract, which is only due to the method used to obtain it in a 1:1 ratio. Obtaining the systems did not adversely affect the content of the active compound.

These results underscore the stability of the anthocyanin content, particularly pelargonidin-3,5-glycoside, and its resilience to formulation processes. The utilization of mass ratios in the formulation highlights the successful preservation of anthocyanins during the preparation of these systems. The results of the determination can be found in Table 2. Lyophilization, also known as freeze-drying, has emerged as an exceptionally effective method for preparing formulated systems, especially when dealing with sensitive compounds like anthocyanins. This technique offers distinct advantages that make it particularly suitable for maintaining the integrity and potency of anthocyanins in various formulations. One of the key benefits of freeze-drying is its ability to preserve the structural and chemical attributes of anthocyanins, which are known to be vulnerable to heat and oxidative degradation. The freeze-drying method that was used in our study proved to be effective so as to obtain systems that do not affect the anthocyanin content. The main active compound present in the raw material is pelargonidin-3,5-glucoside. The content of this compound per milligram of lyophilizate was 9.94 ± 0.28, which is twice as high as the content determined in the systems. Another freeze-drying process to obtain the systems will not adversely affect the content of active compounds. Pomegranate flowers are a valuable raw material rich in anthocyanins. Thanks to the high content of this group of compounds, the raw material exhibits health-promoting effects such as anti-diabetic activity [50,51]. Lyophilization is a widely employed method in various industries, such as pharmaceuticals, particularly in the creation of amorphous dispersion systems and for encapsulation purposes [52,53]. Tatasciore et al. [54] used a freeze-drying microencapsulation technique using hops extract. The results of this study demonstrate the feasibility of lyophilization in the development of functional ingredients, offering new perspectives for hop applications in the food and non-food sectors. The encapsulation of active compounds from Melissa officinalis leaves in β-cyclodextrin and modified starch was equally successful [55]. The effectiveness of other cyclodextrins in the encapsulation process was demonstrated in a review paper by Li et al. [56].

### 3.2. FT-IR Analysis

The FTIR spectrum provides a fingerprint-like pattern of absorption peaks, each corresponding to specific functional groups in the sample. By analyzing these peaks and their positions, it is possible to identify the functional groups and chemical bonds present in the sample. The purpose of the study was to confirm the presence of interactions between pelargonidin-3,5-glucoside and the substances used to form the systems. The presence of such bonds can translate into properties such as a stability or release profile. In the context of PL, FTIR analysis was performed on the powdered sample. Detailed assignments of functional groups to the recorded FT-IR bands are summarized in Table S1 (Supplementary Materials). Similar FT-IR spectra of pomegranate flower extract are reported [57,58].

#### 3.2.1. HP-γ-cyclodextrin System (HP-γ-CD/PL)

**Figure 4:**
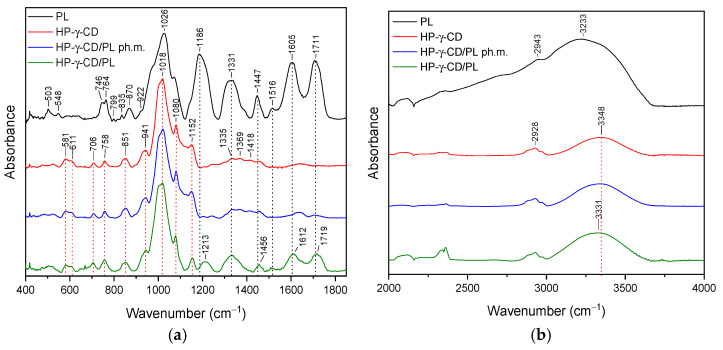
The FT-IR spectra: lyophilized pomegranate extract (PL, black), HP-γ-CD/PL system (HP-γ-CD red), HP-γ-CD/PL physical mixture (HP-γ-CD/PL ph.m., blue), (HP-γ-CD/PL, green)—(**a**) range 400–1800 cm^−1^; (**b**) 2000–4000 cm^−1^.

The most characteristic peaks of HP-γ-CD in the range of ~580–850 were at 581 cm^−1^, 611 cm^−1^, 706 cm^−1^, 758 cm^−1^, 851 cm^−1^ are reported in Supplementary Materials (Table S2). The FT-IR spectra were illustrated in Figure 4a,b with a green line. In the case of the HP-γ-CD/PL system, the typical peaks of both HP-γ-CD and PL with significant changes were identified. Comparing the spectra of HP-γ-CD with the HP-γ-CD/PL system in the range of 400–1800 cm^−1^, we do not observe band shifts; however, we do observe a change in the shape and/or intensity of the bands at 758 cm^−1^, 941 cm^−1^, 1152 cm^−1^. Compared to the physical mixture, the spectrum of the system in the 1200–1800 cm^−1^ range is obscured by PL-specific bands. These bands have a reduced intensity compared to pure PL. Furthermore, the bands observed in pure PL at 1186 cm^−1^, 1331 cm^−1^, 1447 cm^−1^, 1605 cm^−1^, 1711 cm^−1^ in system are shifted to 1213 cm^−1^, 1456 cm^−1^, 1612 cm^−1^, and 1719 cm^−1^, respectively. In the range of 3000–3750 cm^−1^, the band at 3348 cm^−1^ (HP-γ-CD) shifted to 3331 cm^−1^ (HP-γ-CD/PL).

#### 3.2.2. α-Cyclodextrin System (α-CD/PL)

**Figure 5:**
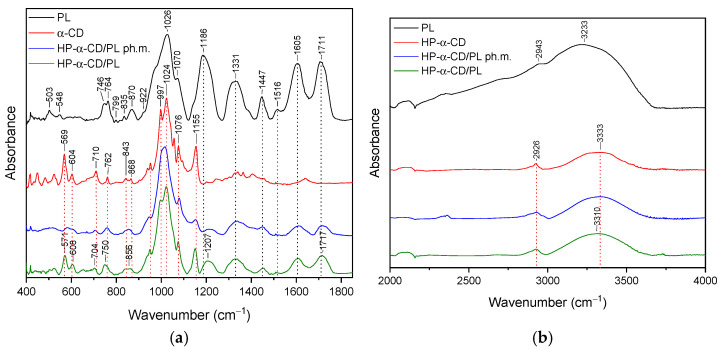
The FT-IR spectra: lyophilized pomegranate extract (PL, black), HP-α-CD/PL system (HP-α-CD red), HP-α-CD/PL physical mixture (HP-α-CD/PL ph.m., blue), (HP-α-CD/PL, green)—(**a**) range 400–1800 cm^−1^; (**b**) 2000–4000 cm^−1^.

In the range of ~570–870 cm^−1^, the most characteristic peaks of α-CD were at 569 cm^−1^, 604 cm^−1^, 710 cm^−1^, 762 cm^−1^, 843 cm^−1^, 868 cm^−1^, 939 cm^−1^, and 951 cm^−1^. Peaks are reported in Supplementary Materials (Table S2). The FT-IR spectra were illustrated in Figure 5a,b, green line In the case of the α-CD/PL system (Figure 5a,b, green line), typical peaks of both α-CD and PL with significant changes were identified. Comparing the spectra of α-CD with α-CD/PL system in the range of 400–1800 cm^−1^, peaks at 569 cm^−1^, 604 cm^−1^, 710 cm^−1^, and 762 cm^−1^ shifted to 571 cm^−1^, 608 cm^−1^, 704 cm^−1^, 750 cm^−1^, respectively. The 1200–1800 cm^−1^ range is obscured by PL-specific bands. These bands have a reduced intensity compared to pure PL.

#### 3.2.3. Methyl-β-cyclodextrin System (Me-β-CD/PL)

**Figure 6:**
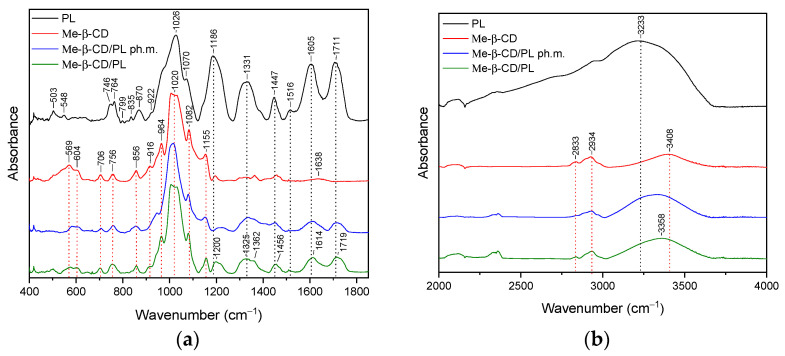
The FT-IR spectra: lyophilized pomegranate extract (PL, black), Me-β-CD/PL (Me-β-CD/PL), Me-β-CD/PL physical mixture (Me-β-CD/PL ph.m., blue), (Me-β-CD/PL, green)—(**a**) range 400–1800 cm^−1^; (**b**) 2000–4000 cm^−1^.

The FT-IR spectra of pure Me-β-CD were illustrated in Figure 6a,b, with a green line. Peaks are reported in the Supplementary Materials (Table S2).

In the case of the Me-β-CD/PL system (Figure 5a,b, green line), typical peaks of both Me-β-CD and PL with significant changes were identified. Comparing the spectra of Me-β-CD with the Me-β-CD/PL system in the range of 400–1800 cm^−1^ we do not observe band shifts; however, we do observe a change in the shape and/or intensity of the bands at 569 cm^−1^, 604 cm^−1^, 706 cm^−1^, 756 cm^−1^, 856 cm^−1^, 916 cm^−1^, 964 cm^−1^, and 1155 cm^−1^. In the range of 1200–1800 cm^−1^, PL-specific bands were observed. These bands are shifting and have a reduced intensity compared to pure PL. In particular, the band observed at 1186 cm^−1^, 1331 cm^−1^, 1447 cm^−1^, 1605 cm^−1^, and 1711 cm^−1^ were shifted to 1200 cm^−1^, 1325 cm^−1^, 1456 cm^−1^, 1614 cm^−1^, and 1719 cm^−1^, respectively. In the range of 3000–3750 cm^−1^, the band at 3408 cm^−1^ (Me-β-CD) shifted to 3358 cm^−1^ (Me-β-CD/PL).

#### 3.2.4. Inulin System (Inu/PL)

**Figure 7:**
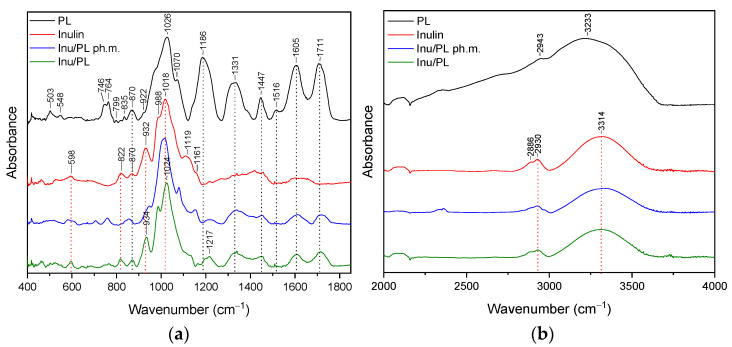
The FT-IR spectra: lyophilized pomegranate extract (PL, black), Inulin (Inu, red), Inu/PL physical mixture (Inu/PL ph.m., blue), Inu/PL system (Inu/PL, green)—(**a**) range 400–1800 cm^−1^; (**b**) 2000–4000 cm^−1^.

Peaks of inulin are reported in the Supplementary Materials (Table S2) and interpreted on the basis of the literature. The FT-IR spectra were illustrated in Figure 7a,b, with a green line.

In the case of the Inu/PL system (Figure 7a,b, green line), typical peaks of both Inu and PL with significant changes were identified. Comparing the spectra of Inu with the Inu/PL system in the range of 400–1800 cm^−1^, band shifts are observed for peaks at 932 cm^−1^ and 1018 cm^−1^. These peaks in the Inu/PL system were observed at 934 cm^−1^ and 1024 cm^−1^. In the range of 1200–1800 cm^−1^, PL-specific bands with reduced intensity were observed (Figure 6a, green line). In addition, the band at 1186 cm^−1^ shifted to 1217 cm^−1^ and had a reduced intensity and a changed shape compared to pure PL. In the range of 3000–3750 cm^−1^, the band at 3314 cm^−1^ (Inu) does not shift in the spectrum of the Inu/PL system.

#### 3.2.5. Arabic Gum System (AGu/PL)

**Figure 8:**
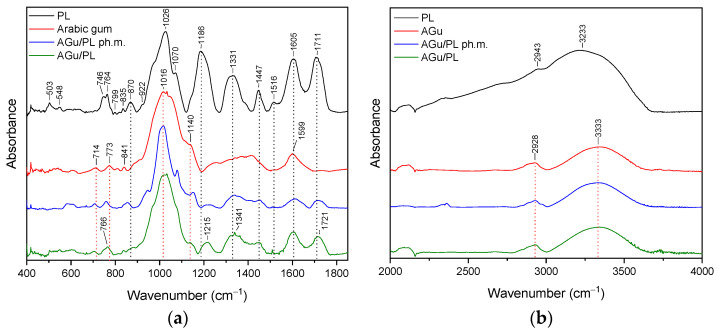
The FT-IR spectra: lyophilized pomegranate extract (PL, black), Arabic gum/PL system (AGu red), Arabic gum/PL physical mixture (AGu/PL ph.m., blue), Arabic gum/PL (AGu/PL, green)—(**a**) range 400–1800 cm^−1^; (**b**) 2000–4000 cm^−1^.

The most characteristic bands in the FT-IR spectra of Arabic gum (AGu) are observed at about 500–900 cm^−1^, 900–1200 cm^−1^, 1400 cm^−1^, 1599 cm^−1^, 2928 cm^−1^, and 3333 cm^−1^ [59]. Peaks are reported in Supplementary Materials (Table S2) based on the literature [41,42]. The FT-IR spectra were illustrated in Figure 8a,b, with a green line.

In the case of the Agu/PL system (Figure 8a,b), the typical peaks of both AGu and PL with significant changes were identified. Comparing the spectra of AGu with AGu/PL system peaks at 1186 cm^−1^, 1331 cm^−1^, and 1711 cm^−1^, shifted to 1215 cm^−1^, 1341 cm^−1^, and 1721 cm^−1^, respectively. In addition, bands of PL observed at 500–800 cm^−1^, and 1070 cm^−1^, disappeared, whereas bands at 1186 cm^−1^, 1331 cm^−1^, 1447 cm^−1^, 1516 cm^−1^, 1605 cm^−1^, and 1711 cm^−1^ have reduced intensity. Shape changes concern the bands of AGu observed in pure samples at 773 cm^−1^ and 1016 cm^−1^. In the 3000–3750 cm^−1^ range, no significant changes in the nature of the AGu/PL system spectrum are observed.

The results of the FT-IR analysis indicate that in the case of HP-γ-CD/PL, Me-β-CD/PL, and AGu/PL, strong intermolecular bonds are formed. The changes observed in the FT-IR spectrum of HP-γ-CD/PL mainly concern the bands corresponding to the C=O (1711 cm^−1^, 1605 cm^−1^), CH2 (1447 cm^−1^), CH3 (1331 cm^−1^) and C-N (1186 cm^−1^) groups characteristic of PL. The shifts in these bands suggest that the indicated groups can form hydrogen bonds with HP-γ-CD. The same is true for Me-β-CD/PL, AGu/PL. This is possible because the literature indicates that active compounds present in plant extracts can form hydrogen bonds with various carriers [60]. However, for α-CD/PL and Inu/PL, no significant differences in the nature of the FT-IR spectra between the physical mixture and the complex are observed, which indicates very weak or no interactions. Strong bonds created between PL and HP-γ-CD, Me-β-CD, and AGu translated into a higher thermal stability of the Pe-3,5-glu (see Section 3.4).

### 3.3. Dissolution Study

**Figure 9:**
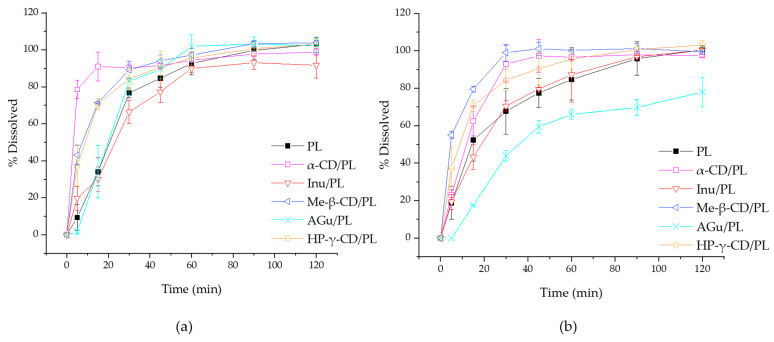
The dissolution rate of lyophilized pomegranate extract and systems in medium at pH = 1.2 (**a**) and at pH = 6.8 (**b**).

The analysis conducted in this study focuses on the release profiles of different substances in relation to the lyophilizate carrier (PL). The analysis aimed to understand how different substances interact with the carrier and what similarities and differences in the release processes may exist depending on the type of substance and the carrier/substance system. The dissolution profile in the study presented here allows us to predict the effect of combinations with prebiotic substances. The addition of excipients can significantly alter the way the active ingredient is released, as many studies have shown. A medium of pH = 1.2 and pH = 6.8 was selected for the release study, corresponding to the environmental conditions of the stomach and small intestine, respectively. The results of the release test are shown in Figure 9. The difference coefficient (*f*_1_) and similarity coefficient (*f*_2_) values were estimated for PL and the obtained systems to evaluate statistical differences between release profiles. The estimated *f*_1_ and *f*_2_ values for PL and the different systems in pH 1.2 are summarized in Table 3. It was observed that the *f*_2_ values for HP-γ-CD/PL, α-CD/PL and Me-β-CD/PL were less than 50, and the *f*_1_ values were greater than 15 for the same samples, suggesting that the dissolution curves of these systems are statistically different. For pH = 6.8 conditions, it was observed that the *f*_2_ values for HP-γ-CD/PL, α-CD/PL, Me-β-CD/PL and AGu/PL were less than 50, and the f_1_ values greater than 15 for α-CD/PL, Me-β-CD/PL, AGu/PL suggesting that the dissolution curves of these systems are statistically different (Table 4).

The findings of the dissolution study at pH 1.2 have revealed a notable pattern: the formulations containing inulin (Inu/PL) and gum Arabic (AGu/PL) exhibit a significant similarity in their drug release profiles compared to the reference formulation—pure lyophilizate. This suggests that the rate at which the active ingredient is released from these two formulations is quite similar, with minimal differences between them. On the other hand, the other formulations tested show distinct variations in their release profiles. Interestingly, formulations that include cyclodextrins, such as HP-γ-CD and Me-β-CD and α-CD, demonstrate a faster release of the active compounds compared to the reference formulation. Observation suggests that cyclodextrins might enhance the speed at which the active ingredients are released. This insight hints at the potential for cyclodextrins to expedite active compound liberation. In the pH 6.8 study, the release profiles for cyclodextrins continue to show that their combination releases the active compound faster. In the case of the AGu/PL system, it is clear that the release profile indicates a lower release rate. In contrast to pH 1.2, acacia gum released less of the substance, which can be explained by its other gelling properties at this pH. In a more neutral environment, such as pH 6.8 (which is more similar to intestinal pH), acacia gum can form more stable gels or matrices. In this case, the release of the active ingredient can be more controlled and delayed, which can lead to a longer and more uniform release. In the context of the release study, the most interesting finding is that formulations containing cyclodextrins, such as HP-γ-CD, Me-β-CD and α-CD, show significantly faster release rates of the active ingredients compared to the reference formulation. This suggests that cyclodextrins may play an important role in accelerating the release of active ingredients from the drug formulation. This acceleration may be due to their ability to form complexes with the active ingredient, which increases its solubility and facilitates release. Testing at pH 6.8, more similar to intestinal conditions, confirms that cyclodextrins still accelerate the release of active ingredients. In the case of acacia gum, a more controlled and delayed release of active ingredients was observed, which is understood to be the result of its ability to form stable gels or matrices in a more neutral environment. Cyclodextrins are often used compounds to improve the solubility of active compounds of plant origin [19,61]. The findings suggest that faster release of active ingredients from cyclodextrin-containing formulations may be beneficial for extract delivery. For patients with diabetes, controlling blood glucose levels after a meal is a key goal to prevent sudden glucose spikes, which can lead to complications. On the other hand, a more controlled release of active ingredients from formulations containing acacia gum can be useful for long-term effects. Such systems can be used to ensure stable glucose levels for longer periods, which is important in maintaining proper diabetes control throughout the day.

### 3.4. Thermal Degradation Kinetic Studies

**Figure 10:**
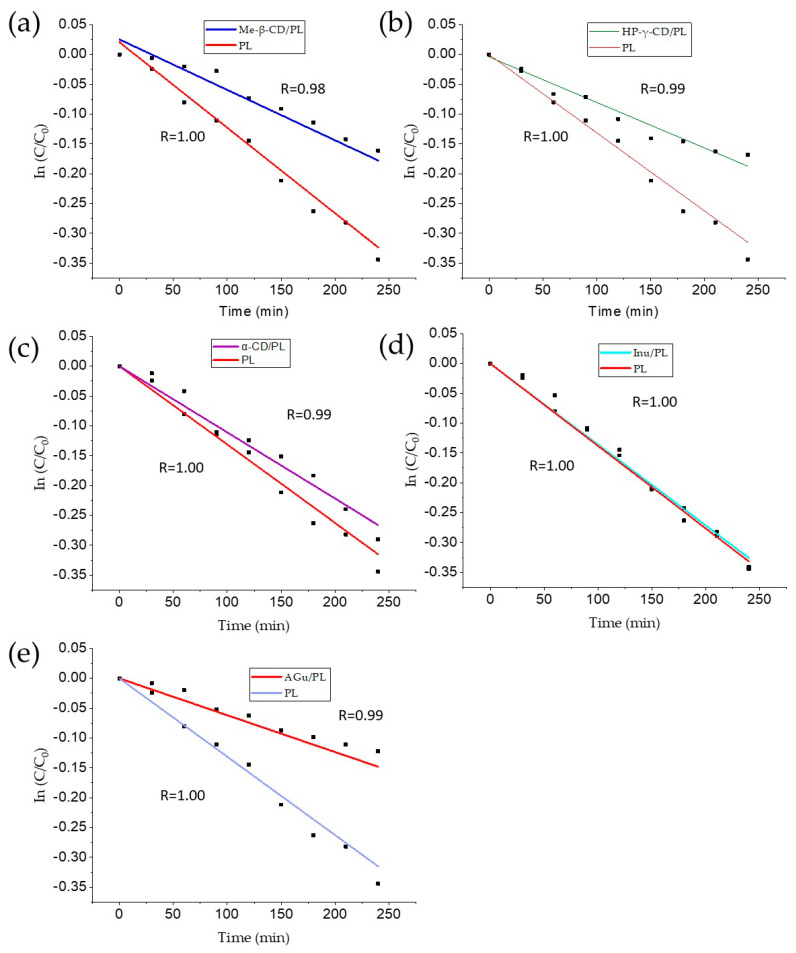
Thermal degradation kinetics of Pe-3,5-Glu in PL and systems (**a**) HP-γ-CD/PL, (**b**) α-CD/PL, (**c**) Me-β-CD/PL, (**d**) Inu/PL, (**e**) AGu/PL with Pearson coefficient (R).

The thermal degradation of Pe-3,5-Glu derived from pomegranate flower extract in the lyophilizer and systems was tracked over time and shown in Figure 10. The reaction rate constant (k) was determined from the slope of the curve. The degradation process of Pe-3,5-Glu anthocyanins during thermal treatment followed the principles of first-order reaction kinetics. The high coefficient of determination (R^2^) indicated the stability of the degradation trends in the systems and lyophilizate, confirming their agreement with the first-order kinetics model. The results are summarized in Table 5. These results are consistent with previous studies involving lyophilized samples and different experimental conditions. The stability of the system was significantly enhanced through the incorporation of methyl-β-cyclodextrin, HP-γ-cyclodextrin, and gum Arabic. These additives played a crucial role in improving the overall stability of the system under investigation. Methyl-β-cyclodextrin, known for its ability to form inclusion complexes, demonstrated notable effectiveness in stabilizing the system. The molecular encapsulation provided by methyl-β-cyclodextrin contributed to the prevention of undesirable interactions and aggregation within the system, leading to enhanced stability. HP-γ-cyclodextrin, with its modified structure, also proved to be an effective stabilizer. The hydroxypropyl groups in HP-γ-CD contributed to increased solubility and improved molecular compatibility, thereby reducing the likelihood of phase separation or degradation, resulting in enhanced stability. Furthermore, the inclusion of gum Arabic, a natural polysaccharide, played a significant role in stabilizing the system. Gum Arabic possesses both hydrophilic and steric stabilizing properties, creating a protective barrier around the system components [62]. In the case of α-cyclodextrin, where a slight difference in improvement in stability was observed, the difference in structure compared to the other cyclodextrins used may result in lower efficiency in forming stable inclusion complexes with anthocyanins. This, in turn, may affect the ability of α-cyclodextrin to protect anthocyanins from degradation. This is due to the differences in the conformation and size of the two types of cyclodextrins, which may affect their ability to form stable complexes with different types of compounds.

The findings by Das et al. [63] demonstrated that anthocyanins follow first-order kinetics during degradation at elevated temperatures, and this degradation process accelerates with an increase in the temperature of exposure. Our results align with prior investigations involving lyophilized samples under varying experimental conditions. Notably, combinations with polysaccharides have proven to be effective in enhancing the stability of anthocyanins at elevated temperatures. Techniques such as microencapsulation or spray drying have been employed for this purpose [54,64]. Cyclodextrins emerge as valuable agents in bolstering anthocyanin stability. Through the formation of inclusion complexes with anthocyanins, cyclodextrins, with their ring-like structure, act as a protective barrier. This protective role prevents anthocyanins from encountering factors that might contribute to their degradation, particularly temperature fluctuations. This property is widely used in strategies to improve the solubility of oral drugs [65]. Currently, nano-delivery vehicles for anthocyanins commonly include polysaccharides, liposomes, and proteins, as highlighted by Jiang and Zhang [66]. Review articles underscore the significance of polysaccharide-based formulations including nanocarriers in the development of anthocyanin-containing products [67]. In a study conducted by da Silva et al. [68] on *Syzygium cumini* extract, carriers with high anthocyanin retention and stability during storage that can be produced using gelation and freeze-drying methods were found. Furthermore, the utilization of beta-cyclodextrin and maltodextrin as encapsulating agents for juçara extract has shown notable benefits. This includes significant thermal stability and enhanced light stability of anthocyanins, along with an expansion of their color intensity across a broader pH range.

### 3.5. Anti-Diabetic and Antioxidant Activity of Lyophilizate and Systems

#### 3.5.1. Results of In Vitro Studies

The analysis of the provided summary of the results of various experiments evaluating antioxidant activity and anti-diabetic potential associated with four parameters: DPPH, CUPRAC, α-glucosidase inhibition and α-amylase inhibition (Table 6). Data indicate that freeze-dried pomegranate flower extract (PL) shows strong activity values in each of these parameters. Significantly, the activity values in these formulations are consistently twice as low as the original PL values, which is expected given the 1:1 dilution. This means that the prebiotic additives did not affect activity, causing only a reduction consistent with the dilution ratio. The values in these formulations remain close to each other, indicating that the included prebiotic substances did not significantly affect activity levels. In this case, although the prebiotic additives may affect certain characteristics of the extract, such as its structure or solubility, they did not significantly affect the extract’s ability to exhibit antioxidant properties and anti-diabetic potential. This means that although some parameters of the extract may have changed under the influence of the prebiotic additives, the anti-diabetic and antioxidant parameters remained stable. Such results of the study may indicate that prebiotic additives interact in a selective manner, involving certain mechanisms or aspects of the extract, but do not affect the properties that were studied. Certainly, such a favorable result has important implications. The demonstration that prebiotic additives did not significantly affect the studied antioxidant and anti-diabetic properties of pomegranate flower extract opens new perspectives for the use of these additives in the future. This means that there is potential for innovative formulations that combine the benefits of prebiotics with the health values of plant extracts.

Scientific research on the antioxidant and anti-diabetic activity of pomegranate flowers has revealed the presence of compounds that exhibit strong antioxidant and antidiabetic properties. Based on scientific publications, the antioxidant activity of pomegranate flowers has been attributed to the presence of various constituents such as anthocyanins whose high content confirmed this study. Additives to active substances can have various uses—they can both affect the activity of the active substance by influencing the solubility of the active compound, for example, and thus improving its activity, or exhibit their own activity. Systems with cyclodextrins significantly improved, among other things, the solubility of compounds of natural origin thus improving their activity [19]. In the publication below, the addition of substances does not improve antioxidant and anti-diabetic properties due to the fact that the extract under study shows very good solubility and the benefits of using auxiliary substances may be present, for example, in improved resistance of the resulting systems to external conditions. Also, the possibility that in some cases the substances may have the effect of improving antioxidant and anti-diabetic properties cannot be completely negated. When considering other mechanisms of action, some scientific publications prove the beneficial effects of these additives. The review by Salema et al. [69] concludes that Arabic gum supplementation reduces blood sugar levels and HbA1C in prediabetic and diabetic rats through hypolipidemic, anti-inflammatory, and antioxidant mechanisms. Additionally, Arabic gum’s potential to stimulate insulin secretion contributes to its hypoglycemic effect. Clinical trials also show significant efficacy of Arabic gum in improving serum glucose levels, highlighting its potential as a dietary intervention for diabetes management [70,71]. Some studies also report that cyclodextrins show inhibitory activity on amylase [72]. In our study, the addition of cyclodextrins did not affect anti-diabetic activity. However, it should be noted that the concentrations tested are not high. Inulin, on the other hand, can help control diabetes and its complications by improving glycemic and lipid parameters and helping with weight control. The precise mechanisms through which inulin leads to weight reduction are not completely clear. It is possible that certain gut hormones like GLP-1, PYY, and ghrelin might contribute to the weight reduction effects associated with inulin consumption [73].

#### 3.5.2. Results of In Silico Studies

**Figure 11:**
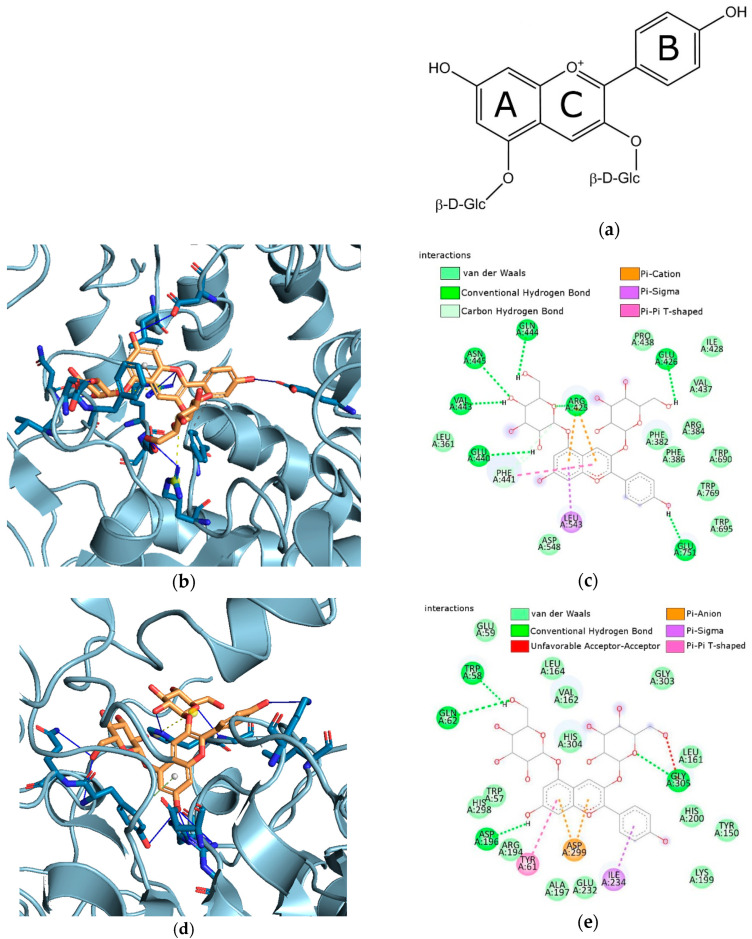
(**a**) Structure of pelargonidin-3,5-glucoside (**b**) Active site gorges of α-glucosidase (PDB id: 4J5T); (**c**) Proposed binding mode of Pe-3,5-glu with α-glucosidase; (**d**) Active site gorges of α-amylase (PDB id: 1OSE); (**e**) Proposed binding mode of Pe-3,5-glu with α-amylase.

Molecular docking has been used to explore the binding of Pe-3,5-glu (Figure 11a) with α-glucosidase and α-amylase. The active site gorges of α-glucosidase (PDB id: 4J5T) and α-amylase (PDB id: 1OSE) were shown in Figure 11b,d. The interactions detected between the Pe-3,5-glu and α-glucosidase were visualized in Figure 11c and with α-amylase in Figure 11e.

The estimated free energy of binding (G) and estimated inhibition constant (*K_i_*) were G = −12.56/G = −12.14 and *K_i_* = 625.63 pM/*K_i_* = 1.26 nM for Pe-3,5-glu with α-glucosidase and α-amylase, respectively. The hydroxyl groups of Pe-3,5-gly binds to Asn445, Gln444, Glu426, Glu440, Glu751, and Val443 of α-glucosidase through conventional hydrogen bonds. Pi-cation, Pi-Sigma and Pi-Pi T-shaped interactions occur between Pe-3,5-gly and Arg425, Phe441, and Leu543, respectively (see Figure 11c). As shown in Figure 11e, the hydroxyl groups of Pe-3,5-gly binds to Asp196, Gln62, Gly305, and TRP58 of α-amylase through conventional hydrogen bonds. The A-ring and C-ring of Pe-3,5-gly form Pi-anion contact with Asp299. Whereas, Pi-Sigma and Pi-Pi T-shaped interactions occur between the B-ring of Pe-3,5-gly and Ile234, and the A-ring of Pe-3,5-gly and Tyr61, respectively.

Molecular docking is a valuable technique used to confirm interactions between enzymes. The latest literature reports show that molecular docking is crucial for discovering new compounds for medical use, predicting interactions between ligands and targets, and comprehending the relationships between structure and activity [74,75]. In silico study of anthocyanidins and anthocyanins, activity has been of interest in various biomedical applications [4,76,77,78,79]. Through molecular docking simulations, researchers have identified favorable inhibitory interactions between anthocyanins and the active sites of enzymes like α-glucosidase and α-amylase [4,76,78,79]. To date, researchers have conducted molecular docking studies to explore the binding interactions of delphinidin-3-glucoside, delphinidin-3-galactoside, delphin, cyanidin, cyanidin-3-glucoside, cyanidin-3-O-glucoside, cyanidin-3,5-glucoside, cyanidin-3-rutinoside, and peonidin-3-glucoside. The findings from these research studies have given a better understanding of how these compounds inhibit enzymes, underscoring the significance of molecular docking in comprehending interactions between enzymes and inhibitors. Our study is the first to explore the binding interactions of Pe-3,5-glu and α-glucosidase/α-amylase enzymes. Based on the estimated free energy of binding (G) and estimated inhibition constant (Ki) for the Pe-3,5-glu/α-glucosidase and Pe-3,5-glu/α-amylase, suggested that Pe-3-glu may be more potent against glucosidase than amylase. The in silico results are consistent with the in vitro findings. The most recent literature affirms the legitimacy of integrating theoretical techniques with in vitro methods. The results of research conducted by Studzińska et al. [75,80] confirmed that the theoretical models aimed at determining the activity of caperatic acid and atranorin in relation to AChE and BChE (cholinesterase enzymes) were consistent with the in vitro results. Promyos et al. [4] indicate that molecular docking allows to correlate the differences in the structure of anthocyanins with their glucosidase-inhibiting effect. Chen et al. [79] by combining in silico and in vitro methods, identified bonds that play an essential role in the binding of α-glucosidase and cyanidin.

### 3.6. Microbiology Study

In studies on prebiotic activity in the context of diabetes, all of the substances mentioned, including hydroxypropyl γ-cyclodextrin, alpha-cyclodextrin, methyl-β-cyclodextrin, inulin, and gum Arabic, showed positive prebiotic activity. Information on the intensity of bacterial growth is provided in Table 7. Research on stimulating the growth of lactic bacteria, particularly *Lactobacillus acidophilus*, *Lactobacillus casei*, *Lactobacillus plantarum*, *Lactobacillus brevis*, and *Lactobacillus rhamnosus* GG, used pomegranate extract as the main research ingredient. Studies have shown that pomegranate extract alone has the ability to inhibit the growth of these bacteria. However, it is interesting to note that combining pomegranate extract with prebiotic substances negated this growth inhibition effect (Table 8). This finding suggests that prebiotic substances may affect the interaction between pomegranate extract and lactobacilli, which reduces the extract’s ability to inhibit bacterial growth in the context of diabetes. Conducting a test for stand-alone substances reiterated their prebiotic potential. These findings are significant and may indicate the potential for using combinations between pomegranate extract and prebiotic substances in therapy or support for people with diabetes, due to their ability to regulate the growth of lactic bacteria in the gut.

Creating systems with prebiotics is a good way to improve the properties of plant-derived substances [17]. The introduction of these substances with prebiotic activity into the diets of people with diabetes may be a promising area of investigation for further research and development of nutritional therapy. The study by Bock et al. [81], provides valuable insights into the impact of prebiotics on metabolic outcomes in individuals with diabetes. The study conducted by Cai et al. [82] focused on investigating the complexation of ferulic acid, caffeic acid, gallic acid, isoquercetin, astragalin, and hyperin with sweet potato starch (SPS). The resulting polyphenol–SPS complexes demonstrated significant potential for application as prebiotics, showcasing the dual beneficial effects on the gut microbiota. Our study found that pomegranate flower extract on its own showed no prebiotic activity and even inhibited the growth of prebiotic bacteria. The literature shows that anthocyanins exhibit prebiotic activity by stimulating the growth of probiotic bacteria, which was not demonstrated in our study. However, anthocyanins are not the only active compounds of the extract. Research conducted by Yisimayili et al. [83] has shown that in pomegranate extract, one of the groups of compounds is gallic acid derivatives. Phenolic acids do not always show prebiotic activity and, on the contrary, can inhibit the growth of bacteria including prebiotic bacteria. The study by Chen et al. [84] suggests that phenolic compounds may have an inhibitory effect on the growth and viability of lactic acid bacteria. This study is relevant to the impact of phenolic acids on *Lactobacillus* and supports the claim that certain phenolic compounds may hinder the growth of lactic acid bacteria. Most of the studies highlight the fact that the effect of polyphenolic compounds depends on polyphenol type, form, dose and strain dependence [85]. The prebiotic activity of the substances used in this publication is confirmed by other studies. α-Cyclodextrin alters the gut microbiota and decreases fat accumulation in obese mice consuming a high-fat diet, which has been demonstrated in the Nihei et al. study [86]. Ren et al.’s study found that supplementing with γ-cyclodextrin in dogs led to a quadratic decrease in fecal *Clostridium perfringens*, indicating a potential impact on microbial ecology. Zhu et al.’s [87] study suggests that the use of cyclodextrins, particularly a combination of α-CD, β-CD, and γ-CD, holds promise as a multifaceted strategy to prevent obesity by modulating energy expenditure, lipid metabolism, and gut microbiota composition in a high-fat diet-fed mouse model.

## 4. Conclusions

In conclusion, the findings from this study underscore the promising potential of combining pomegranate flower extract with prebiotic substances in the treatment of diabetes. Both in vitro and in silico studies have provided substantial evidence supporting the anti-diabetic properties of this combination. The utilization of lyophilization as an extraction method demonstrated its efficacy without compromising the anthocyanin content. The prebiotic activity of the formulated systems was validated through their positive impact on lactobacilli. Furthermore, FT-IR analysis elucidated the intricate relationship between excipients and active substances. The evaluation of the release profiles under different pH conditions revealed noteworthy insights. Inulin was found to have no significant impact on the release mechanism, while cyclodextrins exhibited an accelerating effect. In contrast, Arabic gum displayed the ability to create a prolonged release profile. One of the significant advantages of the formulated systems lies in the enhanced stability observed, particularly in the cases of methyl-β-cyclodextrin, HP-γ-cyclodextrin, and gum Arabic systems. This improvement in stability suggests that these formulations could offer potential advantages in terms of shelf life and practical applications in diabetes treatment.

## Figures and Tables

**Table 1:** Parameters used to create a grid box in the case of α-glucosidase and α-amylase.

	PDB Code	Coordinates of Grid Box	Size of Grid Box (Å)	Maximum Radius Limit (Å)
α-glucosidase	4J5T	x = −10.314 y = −24.456 z = 0.685	x = 126 y = 100 z = 126	0.375
α-amylase	1OSE	x = 35.355 y = 37.073 z = 1.411	x = 114 y = 78 z = 70	0.375

**Table 2:** Content of pelargonidine-3,5-diglucoside in individual systems.

	Content
PL	9.94 ± 0.28 µg/mg
HP-γ-CD/PL	5.05 ± 0.25 µg/mg
α-CD/PL	4.85 ± 0.25 µg/mg
Me-β-CD/PL	4.89 ± 0.29 µg/mg
Inu/PL	5.04 ± 0.20 µg/mg
AGu/PL	5.00 ± 0.43 µg/mg

**Table 3:** Calculated coefficient of difference (*f*_1_) and similarity (*f*_2_) at pH = 1.2.

	*f*_1_	*f*_2_
HP-γ-CD/PL	15.51 *	40.45 *
α-CD/PL	30.89 *	23.16 *
Me-β-CD/PL	20.34 *	34.87 *
Inu/PL	10.60	54.17
AGu/PL	6.37	62.69

* Results beyond the range of similarity of release profiles.

**Table 4:** Calculated coefficient of difference (*f*_1_) and similarity (*f*_2_) at pH = 6.8.

	*f*_1_	*f*_2_
HP-γ-CD/PL	10.02	19.80 *
α-CD/PL	15.43 *	43.13 *
Me-β-CD/PL	28.24 *	31.43 *
Inu/PL	3.64	70.37
AGu/PL	32.71 *	31.09 *

* Results beyond the range of similarity of release profiles.

**Table 5:** Kinetic parameters for the degradation.

	k × 10^3^ (min^−1^)	t^1/2^ (h)	R^2^
PL	1.38 ± 0.10	8.85 ± 0.70	0.991
HP-γ-CD/PL	0.80 ± 0.08	14.47 ± 1.43	0.961
α-CD/PL	1.11 ± 0.02	10.42 ± 0.23	0.977
Me-β-CD/PL)	0.63 ± 0.19	18.88 ± 1.15	0.971
Inu/PL	1.36 ± 0.07	8.50 ± 0.42	0.993
AGu/PL	0.53 ± 0.01	19.83 ± 0.39	0.984

**Table 6:** Anti-diabetic and antioxidant activity expressed as IC_50_ value.

	DPPH (IC_50_ mg/mL)	CUPRAC (IC_50_ mg/mL)	α-Glucosidase (IC_50_ µg/mL)	α-Amylase (IC_50_ mg/mL)
PL	0.028 ± 0.004	0.039 ± 0.004	1.77 ± 0.11	0.18 ± 0.01
HP-γ-CD/PL	0.055 ± 0.003	0.087 ± 0.007	3.37 ± 0.07	0.34 ± 0.02
α-CD/PL	0.059 ± 0.002	0.087 ± 0.002	3.47 ± 0.28	0.38 ± 0.02
Me-β-CD/PL)	0.057 ± 0.001	0.079 ± 0.010	3.48 ± 0.29	0.38 ± 0.01
Inu/PL	0.050 ± 0.004	0.090 ± 0.009	3.70 ± 0.46	0.35 ± 0.02
AGu/PL	0.049 ± 0.001	0.076 ± 0.002	3.34 ± 0.25	0.38 ± 0.03
Trolox	0.113 ± 0.002	0.0636 ± 0.001	n.a.*	n.a.*
Acarbose	n.a.*	n.a.*	3246.39 ± 33.1	0.17 ± 0.02

* n.a.—Not applicable.

**Table 7:** Prebiotic activity of tested substances.

		*Lactobacillus acidophilus*	*Lactobacillus casei*	*Lactobacillus plantarum*	*Lactobacillus brevis*	*Lactobacillus rhamnosus GG*	*Lactobacillus reuteri*	*Pediococcus pentosaceus*	*Lactococcus lactis*	*Lactobacillus fermentum*	*Streptococcus thermophilus*
		Bacterial Count (cfu/mL)									
α-CD	Time zero	2.70 × 10^2^	2.00 × 10^2^	2.70 × 10^2^	5.70 × 10^2^	4.60 × 10^2^	1.90 × 10^2^	2.00 × 10^2^	3.80 × 10^2^	1.40 × 10^2^	3.40 × 10^2^
	18 h	7.00 × 10^7^	2.80 × 10^7^	5.60 × 10^8^	2.90 × 10^7^	9.50 × 10^7^	3.50 × 10^8^	5.83 × 10^2^	4.80 × 10^7^	3.20 × 10^7^	8.20 × 10^7^
Inu	Time zero	4.80 × 10^2^	2.00 × 10^2^	2.00 × 10^2^	6.90 × 10^2^	2.70 × 10^2^	5.30 × 10^2^	3.60 × 10^2^	8.30 × 10^2^	1.90 × 10^2^	2.70 × 10^2^
	18 h	3.80 × 10^7^	3.50 × 10^7^	5.40 × 10^7^	3.30 × 10^7^	5.20 × 10^8^	3.00 × 10^3^	3.00 × 10^3^	3.00 × 10^3^	3.00 × 10^3^	3.00 × 10^3^
HP-γ-CD	Time zero	2.70 × 10^2^	4.40 × 10^2^	2.00 × 10^2^	2.10 × 10^2^	2.70 × 10^2^	2.70 × 10^2^	4.40 × 10^2^	2.00 × 10^2^	2.10 × 10^2^	4.40 × 10^2^
	18 h	3.20 × 10^6^	3.70 × 10^2^	5.80 × 10^6^	5.90 × 10^7^	3.00 × 10^3^	3.00 × 10^3^	3.70 × 10^6^	5.80 × 10^6^	5.90 × 10^7^	3.70 × 10^6^
Me-β-CD/	Time zero	9.30 × 10^2^	7.40 × 10^2^	2.70 × 10^2^	2.70 × 10^2^	3.40 × 10^2^	2.70 × 10^2^	2.00 × 10^2^	5.40 × 10^2^	2.30 × 10^2^	2.00 × 10^2^
	18 h	3.40 × 10^7^	2.40 × 10^7^	3.60 × 10^7^	4.92 × 10^2^	5.10 × 10^7^	3.30 × 10^7^	3.10 × 10^6^	3.00 × 10^3^	3.00 × 10^3^	3.00× 10^3^
AGu	Time zero	2.00 × 10^2^	5.30 × 10^2^	1.70 × 10^2^	8.30 × 10^2^	5.60 × 10^2^	6.40 × 10^2^	4.40 × 10^2^	7.70 × 10^2^	2.00 × 10^2^	8.30 × 10^2^
	18 h	0.00	3.00 × 10^3^	3.00 × 10^3^	3.00 × 10^3^	3.30 × 10^6^	3.20 × 10^6^	3.70 × 10^6^	3.00 × 10^3^	3.00 × 10^3^	3.00× 10^3^

**Table 8:** Prebiotic activity of tested systems.

		*Lactobacillus acidophilus*	*Lactobacillus casei*	*Lactobacillus plantarum*	*Lactobacillus brevis*	*Lactobacillus rhamnosus GG*	*Lactobacillus reuteri*	*Pediococcus pentosaceus*	*Lactococcus lactis*	*Lactobacillus fermentum*	*Streptococcus thermophilus*
		Bacterial Count (cfu/mL)									
PL	Time zero	7.70 × 10^2^	3.40 × 10^2^	2.00 × 10^2^	5.70 × 10^2^	9.10 × 10^2^	4.30 × 10^2^	8.10 × 10^2^	3.20 × 10^2^	2.20 × 10^2^	3.40 × 10^2^
	18 h	<10	<10	<10	<10	<10	<10	<10	<10	<10	<10
α-CD/PL	Time zero	2.70 × 10^2^	2.00 × 10^2^	2.70 × 10^2^	5.70 × 10^2^	4.60 × 10^2^	1.90 × 10^2^	2.00 × 10^2^	3.80 × 10^2^	1.40 × 10^2^	3.40 × 10^2^
	18 h	7.00 × 10^7^	2.80 × 10^7^	5.60 × 10^8^	2.90 × 10^7^	9.50 × 10^7^	3.50 × 10^8^	5.83 × 10^2^	4.80 × 10^7^	3.20 × 10^7^	8.20 × 10^7^
Inu/PL	Time zero	4.80 × 10^2^	2.00 × 10^2^	2.00 × 10^2^	6.90 × 10^2^	2.70 × 10^2^	5.30 × 10^2^	3.60 × 10^2^	8.30 × 10^2^	1.90 × 10^2^	2.70 × 10^2^
	18 h	3.80 × 10^7^	3.50 × 10^7^	5.40× 10^7^	3.30 × 10^7^	5.20 × 10^8^	3.00 × 10^3^	3.00 × 10^3^	3.00 × 10^3^	3.00 × 10^3^	3.00 × 10^3^
HP-γ-CD/PL	Time zero	2.70 × 10^2^	4.40 × 10^2^	2.00 × 10^2^	2.10 × 10^2^	2.70 × 10^2^	2.70 × 10^2^	4.40 × 10^2^	2.00 × 10^2^	2.10 × 10^2^	4.40 × 10^2^
	18 h	3.20 × 10^6^	3.70 × 10^6^	5.80 × 10^6^	5.90 × 10^7^	3.00 × 10^3^	3.00 × 10^3^	3.70 × 10^6^	5.80 × 10^6^	5.90 × 10^7^	3.70 × 10^6^
Me-β-CD/PL	Time zero	9.30 × 10^2^	7.40 × 10^2^	2.70 × 10^2^	2.70 × 10^2^	3.40 × 10^2^	2.70 × 10^2^	2.00 × 10^2^	5.40 × 10^2^	2.30 × 10^2^	2.00 × 10^2^
	18 h	3.40× 10^7^	2.40 × 10^7^	3.60× 10^7^	4.92 × 10^2^	5.10 × 10^7^	3.30 × 10^7^	3.10 × 10^6^	3.00 × 10^3^	3.00 × 10^3^	3.00 × 10^3^
AGu/PL	Time zero	2.00 × 10^2^	5.30 × 10^2^	1.70 × 10^2^	8.30 × 10^2^	5.60 × 10^2^	6.40 × 10^2^	4.40 × 10^2^	7.70 × 10^2^	2.00 × 10^2^	8.30 × 10^2^
	18 h	3.00 × 10^3^	3.00 × 10^3^	3.00 × 10^3^	3.00 × 10^3^	3.30 × 10^6^	3.20 × 10^6^	3.70 × 10^6^	3.00 × 10^3^	3.00 × 10^3^	3.00 × 10^3^

## Data Availability

Data are available in a publicly accessible repository.

## Supplementary Materials

The following supporting information can be downloaded at: https://www.mdpi.com/article/10.3390/pharmaceutics16040526/s1, Table S1: S1 FT-IR analysis: selected characteristic bands (in cm^−1^) of pomegranate lyophilizate (PL). Functional group assignment and phytocompounds identified based on literature; Table S2. FT-IR analysis: Characteristic absorption bands (in cm^−1^) of the HP-γ-CD, α-CD, Me-β-CD, Inu, AGu Functional group assignment based on literature.
