# Supplementary material for: Prebiotic Systems Containing Anthocyanin-Rich Pomegranate Flower Extracts with Antioxidant and Antidiabetic Effects
Source: Pharmaceutics. 2024 Apr 10;16(4):526. doi: 10.3390/pharmaceutics16040526 (PMC11054034; doi:10.3390/pharmaceutics16040526)
Supplement: Supplementary file 1 [file pharmaceutics-16-00526-s001.zip › pharmaceutics-2923232-supplementary.pdf]

Supplementary Materials

Table S1. FT-IR analysis: selected characteristic bands (in cm^{-1}) of pomegranate lyophilizate (PL). Functional group assignment and phytocompounds identified based on literature.

Wavelength (cm^{-1})	**Functional group assignment**	**Phytocompounds identified**
870	P-O-C stretching vibration	aromatic phosphates
922	P-O-C stretching vibration	aromatic phosphates
1026	phosphate ion	phosphate compound
1070	phosphate ion	phosphate compound
1186	C-N stretching vibration	amines
1331	CH_3 deformation	alkanes
1447	CH_2 bending	aldehydes and ketones
1516	skeletal vibration of the aromatic ring	-
1605	C=O stretching vibration, ketone group	ketone compound
1711	C=O stretching vibration	carbonyl
2943	O-H stretching vibration, acidic	carboxylic acids
3233	O-H stretching vibration, hydroxy group, H-bonded	poly hydroxy compound

Table S2. FT-IR analysis: Characteristic absorption bands (in cm^{-1}) of the HP-γ-CD, α-CD, Me-β-CD, Inu, AGu Functional group assignment based on literature

**Substance**	**Wavelength (cm^{-1})**	**Functional group assignment**	**Reference**
1. HP-γ-CD	581	O–H and C–H in-plane and out-of-plane bending vibrations, C–C and C–O stretching vibrations	[1]
	611		
	706		
	758		
	851	C–C–H bending vibrations, C–O and C–C stretching vibrations	
	941	skeletal vibration involving α–1,4 linkage	
	1018	O–C–H, C–C–H, and C–C–O bending vibrations	
	1080	C–O and C–C stretching vibrations, C–O–C bending vibrations	
	1152	C–O and C–C stretching vibrations, C–O–C bending vibrations	
	1335	coupled C–C–H, C–O–H, and H–C–H bending vibrations	
	1369	C–H from CH_2 and O–H bending vibrations	
	1418	C–H from CH_2 bending vibrations	
	2928	C–H stretching vibrations	
	3348	O–H stretching vibrations	
2. α-CD	569	O–H and C–H in-plane and out-of-plane bending vibrations, C–C and C–O bond stretching vibrations	[2–5]
	604		
	710		
	762		
	843		
	868		
	939		
	951		
	1024	–C–C–O stretching vibration	
	1076	C–O stretching vibration	
	1155	–C–O–C antisymmetric stretching vibration	

		of the C–O–C glycosidic bridge	
	1200–1500	O–H, C–H and H–C–H bonds	
	1406	bending of –C–H from –CH_2 and bending of O–H	
	1641	O–H oscillating vibration	
	2926	C–H stretching vibration from –CH_2	
	3333	O–H stretching vibration	
3. Me-β-CD	569	framework vibration of α-pyranose	[6–8]
	604	in-plane and out-of-plane bending vibrations of O–H, and C–H bonds, C–C and C–O bond stretching vibrations	
	706		
	756		
	856		
	916		
	964	framework vibration of α–pyranose	
	1020	C–H and C–O stretching vibrations	
	1082		
	1155		
	1200–1500	O–H, C–H and H–C–H bending vibrations	
	1638	crystallized water	
	2833	methoxy (O–CH_3) group stretching	
	2934	C–H stretching vibration	
	3408	O–H stretching vibration	
4. Inu	598	presence of pyranose rings in polymer chain	[9–14]
	822	2-ketose (pyranose or furanose)	
	870	CH_2 ring vibration of β-anomer	
	932	α-D-Glucopyranosyl residue in chain, C–C and C–O stretching, C–O–H and C–O–C deformation modes of oligo- and polysaccharides	
	988	C–C and C–O stretching, C–O–H and C–O–C deformation modes of oligo- and polysaccharides	
	1018	C–O stretching vibrations in the furanose ring	
	1119	presence of ketal groups (C–O–C–O–C), C–O–C stretching vibrations in the furanose ring	
	1200–1400	C–H bending vibrational modes	
	2886	C–H from CH_2 asymmetric stretching vibrations	
	2930	C–H from CH_2 stretching vibrations	
	3314	OH group	
5. AGu	500–900	CCO, COC, symmetrical and asymmetrical ring breathing vibration	[15,16]
	900–1200	fingerprint of carbohydrates	
	~1400	COO–symmetric stretching vibration	
	1599	COO–asymmetric stretching vibration	
	2928	C–H stretching vibration	
	3333	O–H stretching, characteristic of glucosidic ring	

Ad. 1 Peaks are associated with in-plane and out-of-plane bending vibrations of O–H, and C–H bonds as well as C–C and C–O bond stretching vibrations. The most intense bands are recorded at 1018 cm–1 (O–C–H, C–C–H, and C–C–O bending vibrations), 1080 cm−1 (C–O and C–C stretching vibrations, C–O–C bending vibrations), and 1152 cm−1 (C–O and C–C stretching vibrations, C–O–C bending vibrations). Between 1200 cm−1 and 1500 cm−1, peaks at 1335 cm−1, 1369 cm−1 and 1418 cm−1 can be

distinguished (see Figure 3, red line and Table S2). The band at 2928 cm−1 corresponds to stretching vibrations of the C–H bonds. The broad band with a maximum of 3348 cm−1 is associated with the O–H bond stretching vibration.

Ad. 2 Peaks associated with in-plane and out-of-plane bending vibrations of O–H, and C–H bonds as well as C–C and C–O bond stretching vibrations. The most intense bands are recorded at 1024 cm–1, 1076 cm−1, and 1155 cm−1. These are associated with stretching vibrations of the –C–C–O, C–O, and –C–O–C bonds, respectively. Between 1200 cm−1 and 1500 cm−1 a complex sequence of peaks attributed mainly to the bending of the O–H, C–H and H–C–H bonds. The band located at 1641 cm−1 is attributed to the oscillating vibrations of the O–H bonds at the glucose unit at the C4 and C5 carbon and the scissor-like C–H bonds. The band at 2926 cm−1 corresponds to stretching vibrations of the C–H bonds. The broadband with a maximum of 3333 cm−1 is associated with the O–H bond stretching vibration.

Ad. 3 Peaks are characterized by the prominent bands in the range of ~570–870 cm−1 (framework vibration of α-pyranose, in-plane and out-of-plane bending vibrations of O–H, and C–H bonds, C–C and C–O bond stretching vibrations), intense bands between 1000–1200 cm−1 (–C–C–O, C–O, and –C–O–C bonds), complex sequence of peaks at about 1200–1500 cm−1 (O–H, C–H and H–C–H bending vibrations), and three characteristic peaks in the range of 2800–3750 cm−1 (methoxy (O–CH3) group, C–H, and O–H stretching vibrations)

Ad. 4 The FT-IR spectrum of inulin (Inu) shows a lot of absorption bands in the range of ~600–1700 cm−1 and two characteristic peaks in the range of ~2750–3750 cm−1. The peak observed at about 600 cm-1 confirms the presence of pyranose rings in the polymer chain. Bands at 822 and 870 cm−1 are attributed to the = C–H ring vibration in the presence of 2-ketofuranose. Between 900 cm-1 and 1500 cm−1, a complex sequence of peaks attributed mainly to the C—C, C–O stretching and C—O—H, C—O—C deformation vibration of various oligo- and polysaccharides. In this range, the most characteristic bands are at 932 cm−1, 988 cm−1, 1018 cm−1, and 1119 cm−1 (see Table S2). Peaks in the range of 1200–1400 cm−1 arise from the C–H bending vibrational modes. In the literature, the band between 1500 cm−1 and 1700 cm−1 (with a maximum at about 1600 cm−1) is described as non-specific for inulin. This band is attributed to the absorption of water, due to the hygroscopic properties of of this homopolysaccharide. Bands at 2886 cm−1, 2930 cm−1, and 3314 cm−1 were also observed, and corresponding to the C–H from CH2 asymmetric stretching, C–H from CH2 symmetric stretching, and OH group, respectively

Ad. 5 within the range of 500–900 cm−1AGu show weak peaks assigned to CCO, COC, symmetrical and asymmetrical ring breathing vibration. The strong peaks observed at 900–1200 cm−1 are the fingerprints of carbohydrates. At about 1400 cm−1 was peak characteristic to the COO–symmetric stretching. Whereas, a strong peak at 1599 cm−1 corresponds to the COO–asymmetric stretching. The peak at 2928 cm−1 is attributed to the C–H stretching vibration. A broad absorption band at 3333 cm−1 is attributed to the glucosidic ring and might be due to the stretching vibration of O–H.

References

1. Misiuk, W.; Jasiuk, E. Study of the Inclusion Interaction of HP-γ-Cyclodextrin with Bupropion and Its Analytical Application. *Journal of Molecular Structure* **2014**, *1060*, 272–279.
2. Wei, M.; Davis, W.; Urban, B.; Song, Y.; Porbeni, F.E.; Wang, X.; White, J.L.; Balik, C.M.; Rusa, C.C.; Fox, J. Manipulation of Nylon-6 Crystal Structures with Its α-Cyclodextrin Inclusion Complex. *Macromolecules* **2002**, *35*, 8039–8044.
3. Saha, B.C.; Saha, S.; Das, K.; Basak, S.; Roy, M.N. Investigation of Inclusion Complexes of Sodium Valproate Inside Into a and β-Cyclodextrins.

4. Stasiłowicz-Krzemień, A.; Rosiak, N.; Płazińska, A.; Płaziński, W.; Miklaszewski, A.; Tykarska, E.; Cielecka-Piontek, J. Cyclodextrin Derivatives as Promising Solubilizers to Enhance the Biological Activity of Rosmarinic Acid. *Pharmaceutics* **2022**, *14*, 2098, doi:10.3390/pharmaceutics14102098.
5. Ho, B.T.; Joyce, D.C.; Bhandari, B.R. Encapsulation of Ethylene Gas into α-Cyclodextrin and Characterisation of the Inclusion Complexes. *Food Chemistry* **2011**, *127*, 572–580.
6. Yu, J.-G.; Huang, K.-L.; Liu, S.-Q.; Tang, J.-C. Preparation and Characterization of Soluble Methyl-β-Cyclodextrin Functionalized Single-Walled Carbon Nanotubes. *Physica E: Low-Dimensional Systems and Nanostructures* **2008**, *40*, 689–692.
7. Chao, J.; Liu, Y.; Zhang, Y.; Zhang, J.; Zhang, Y.; Guo, Z.; Wang, Y.; Qin, L.; Zhang, B. Investigation of the Inclusion Behavior of Ofloxacin with Methyl-β-Cyclodextrin. *Journal of Molecular Liquids* **2014**, *200*, 404–409.
8. Siva, S.; Li, C.; Cui, H.; Meenatchi, V.; Lin, L. Encapsulation of Essential Oil Components with Methyl-β-Cyclodextrin Using Ultrasonication: Solubility, Characterization, DPPH and Antibacterial Assay. *Ultrasonics Sonochemistry* **2020**, *64*, 104997.
9. Saud, K.T.; Xu, J.; Wilkanowicz, S.; He, Y.; Moon, J.J.; Solomon, M.J. Electrosprayed Microparticles from Inulin and Poly (Vinyl) Alcohol for Colon Targeted Delivery of Prebiotics. *Food Hydrocolloids* **2023**, *140*, 108625.
10. Romano, N.; Araujo-Andrade, C.; Lecot, J.; Mobili, P.; Gómez-Zavaglia, A. Infrared Spectroscopy as an Alternative Methodology to Evaluate the Effect of Structural Features on the Physical-Chemical Properties of Inulins. *Food Research International* **2018**, *109*, 223–231.
11. Petkova, N.T.; Sherova, G.; Denev, P.P. Characterization of Inulin from Dahlia Tubers Isolated by Microwave and Ultrasound-Assisted Extractions. *International Food Research Journal* **2018**, *25*.
12. Akram, W.; Garud, N. Optimization of Inulin Production Process Parameters Using Response Surface Methodology. *Future Journal of Pharmaceutical Sciences* **2020**, *6*, 1–9.
13. Pontes, A.G.O.; Silva, K.L.; da Cruz Fonseca, S.G.; Soares, A.A.; de Andrade Feitosa, J.P.; Braz-Filho, R.; Romero, N.R.; Bandeira, M.A.M. Identification and Determination of the Inulin Content in the Roots of the Northeast Brazilian Species Pombalia Calceolaria L. *Carbohydrate polymers* **2016**, *149*, 391–398.
14. El-Kholy, W.M.; Aamer, R.A.; Ali, A.N.A. Utilization of Inulin Extracted from Chicory (Cichorium Intybus L.) Roots to Improve the Properties of Low-Fat Synbiotic Yoghurt. *Annals of Agricultural Sciences* **2020**, *65*, 59–67.
15. Sheikhzadeh, S.; Alizadeh, M.; Rezazad, M.; Hamishehkar, H. Application of Response Surface Methodology and Spectroscopic Approach for Investigating of Curcumin Nanoencapsulation Using Natural Biopolymers and Nonionic Surfactant. *Journal of food science and technology* **2016**, *53*, 3904–3915.
16. Ibekwe, C.A.; Oyatogun, G.M.; Esan, T.A.; Oluwasegun, K.M. Synthesis and Characterization of Chitosan/Gum Arabic Nanoparticles for Bone Regeneration. *Am. J. Mater. Sci. Eng* **2017**, *5*.
